# Colorectal cancer mortality trends in Serbia during 1991–2010: an age-period-cohort analysis and a joinpoint regression analysis

**DOI:** 10.1186/s40880-016-0118-y

**Published:** 2016-06-22

**Authors:** Milena Ilic, Irena Ilic

**Affiliations:** Department of Epidemiology, Faculty of Medical Sciences, University of Kragujevac, S. Markovica 69, Kragujevac, 34000 Serbia; Faculty of Medical Sciences, University of Kragujevac, S. Markovica 69, Kragujevac, 34000 Serbia

**Keywords:** Colorectal cancer, Mortality, Trend, Joinpoint regression analysis

## Abstract

**Background:**

For both men and women worldwide, colorectal cancer is among the leading causes of cancer-related death. This study aimed to assess the mortality trends of colorectal cancer in Serbia between 1991 and 2010, prior to the introduction of population-based screening.

**Methods:**

Joinpoint regression analysis was used to estimate average annual percent change (AAPC) with the corresponding 95% confidence interval (CI). Furthermore, age-period-cohort analysis was performed to examine the effects of birth cohort and calendar period on the observed temporal trends.

**Results:**

We observed a significantly increased trend in colorectal cancer mortality in Serbia during the study period (AAPC = 1.6%, 95% CI 1.3%–1.8%). Colorectal cancer showed an increased mortality trend in both men (AAPC = 2.0%, 95% CI 1.7%–2.2%) and women (AAPC = 1.0%, 95% CI 0.6%–1.4%). The temporal trend of colorectal cancer mortality was significantly affected by birth cohort (*P* < 0.05), whereas the study period did not significantly affect the trend (*P* = 0.072). Colorectal cancer mortality increased for the first several birth cohorts in Serbia (from 1916 to 1955), followed by downward flexion for people born after the 1960s. According to comparability test, overall mortality trends for colon cancer and rectal and anal cancer were not parallel (the final selected model rejected parallelism, *P* < 0.05).

**Conclusions:**

We found that colorectal cancer mortality in Serbia increased considerably over the past two decades. Mortality increased particularly in men, but the trends were different according to age group and subsite. In Serbia, interventions to reduce colorectal cancer burden, especially the implementation of a national screening program, as well as treatment improvements and measures to encourage the adoption of a healthy lifestyle, are needed.

## Background

It is estimated that colorectal cancer causes over 600,000 deaths worldwide annually, accounting for 7.6% of cancer-related deaths among men and 8.6% among women; this makes colorectal cancer the fourth most common cause of cancer-related death [1–3]. Although the number of deaths from colorectal cancer is almost the same in both developed and developing regions, the death rates vary by more than five times around the world [3]. According to the GLOBOCAN 2008 estimates, the highest colorectal cancer mortalities in both sexes were found in Central and Eastern Europe (20.3 per 100,000 for men, 12.1 per 100,000 for women); the lowest mortalities in both sexes were found in Middle Africa (3.5 per 100,000 and 2.7 per 100,000, respectively) [1–3].

During the last decade of the twentieth century, colorectal cancer mortality declined steeply in more developed regions: western and northern European countries (the United Kingdom, France, and Sweden), countries in North America (the United States of America and Canada), and Australia [3, 4]. At the beginning of the twenty-first century, declines were also observed in Japan, Germany, and Italy [5, 6]. In contrast, most countries in Eastern Europe (such as Russia and Romania) and Southern Europe (such as Portugal and Spain) saw a steep increase in colorectal cancer mortality; however, this was not the case in all countries (e.g., the Czech Republic, Hungary, and Slovakia) [3–5]. Trends were particularly favorable in young people (under 50 years of age), especially in young women [6]. The decreasing mortality trend for colorectal cancer in developed countries after 1990 may be attributable to the implementation of screening programs and improvements in treatment [7, 8]. More recent screening programs implemented in some European countries probably have had some effect on colorectal cancer mortality [9, 10]; recent socio-economic changes may have also had a role in reducing colorectal cancer incidence and mortality [11].

Most patients who died of colorectal cancer were aged 50 years or older [3, 6, 12]. Colorectal cancer mortality was 1–2 times higher for men than for women [6]. The etiology of colorectal cancer has not been entirely elucidated, but older age, inflammatory bowel disease, family history, dietary factors, obesity, low physical activity, alcohol consumption, and tobacco smoking have been recognized as risk factors for colorectal cancer [13, 14].

The purpose of this study was to assess temporal changes in colorectal cancer mortality in Serbia between 1991 and 2010, prior to the introduction of population-based screening.

## Methods

### Data sources

Colorectal cancer was defined according to the International Classification of Diseases (ICD) as malignant neoplasm of the colon (ICD-9 code 153 and ICD-10 code C18) and malignant neoplasm of the rectum, rectosigmoid junction, and anus (ICD-9 code 154 and ICD-10 codes C19–21). Mortality data were obtained from the Statistical Office of the Republic of Serbia (unpublished data). This analysis comprised the entire population of the Republic of Serbia (all ages), during the period 1991–2010, excluding the Autonomous Province of Kosovo and Metohia, for which data have been unavailable since 1998. Data on the population and composition of the Republic of Serbia (approximately 7.5 million people) by sex and age were obtained from the population censuses in 1991 and 2002; for inter-census years, estimates published by the Statistical Office of the Republic of Serbia were used [15]. In Europe, Serbia had the largest number of internally displaced people and refugees (nearly 400,000 people) during the period 1991–2010; these data were included in the Serbian population and could not be set aside as a special contingent. This study was approved by the Ethics Committee of the Faculty of Medical Sciences, University of Kragujevac (protocol: 01-4801).

### Statistical analysis

We calculated three types of death rates (per 100,000 people): crude, age- and sex-specific, and age-standardized. The age-standardized rate (ASR) was calculated by direct method (Segi’s world population was used as standard population, stratified by 10-year age groups) [16]. To obtain the number of deaths expected in the standard population, the specific rates that were observed in Serbia were applied on the standard population using direct method of standardization. The standardized rate in Serbia was calculated by dividing the expected number of deaths in the standard population by the total standard population. The rates, with number of cases, were given for the beginning and the end of the study period for several age groups (30–34, 35–39, …, and 75–79).

Trends in colorectal cancer mortality were assessed using joinpoint regression analysis (Joinpoint Regression Software, Version 4.0.4—May 2013; Statistical Methodology and Applications Branch, Surveillance Research Program of the US National Cancer Institute; Bethesda, MD, USA) according to the method proposed by Kim et al. [17]. Lerman’s Grid Search Method [18] was used to create a fine grid of all possible locations for joinpoints specified by the settings and to determine the sum of squared error at each one. The Grid Search Method was used to fit the segmented line regression where the joinpoint estimates occur at discrete grid points and to determine the best fit for each individual model. The program started with a minimum of zero joinpoint and was tested to determine whether one or more joinpoints were statistically significant (up to the maximum of five joinpoints that were allowed for each model). The Monte Carlo Permutation method with 4499 replicates was used to test significance. Once the line segments were fortified, the annual percent change (APC) was used to describe the trends in the model. In ASRs of colorectal cancer mortality (1991–2010), trends are presented as average annual percentage changes (AAPCs), which are a summary measure over a fixed interval [19]. For each estimate of APC, 95% confidence intervals (CIs) were calculated and used to determine if the APC for each segment differed significantly from zero. Also, we assessed pairwise differences using a comparability test to determine whether the two segmented line regression functions were parallel (test of parallelism) or whether the two mean functions were identical (test of coincidence) [20]. Two-sided *P* values less than 0.05 were considered statistically significant. Joinpoint results were not shown for the subgroups of deceased people younger than 30 years because fewer than five deaths from colorectal cancer occurred in each 5-year age group in any year. We conducted separate analyses for cancer of the colon and the rectum and anus. We analyzed mortalities for men and women separately to account for sex-related differences in the natural history. Given the small number of deceased people younger than 30 years, we limited our analysis to those aged 30–79.

Additionally, using the United States National Cancer Institute statistical web tool according to the method proposed by Rosenberg et al. [21], we conducted an age-period-cohort analysis to examine the effects of birth cohort and calendar period on the observed temporal trends. For age-period-cohort analysis, we used the colorectal cancer mortality data stratified by 5-year age groups (30–34, 35–39, …, and 75–79); 5-year intervals were also used for calendar periods (1991–1995, 1996–2000, …, and 2006–2010) and birth cohorts (1916–1920, 1921–1925, …, and 1976–1980). The central age group, period, and birth cohort were defined as the reference. The degrees of freedom counted the number of free parameters included in each test. The age-period-cohort analysis summarized the deviations from linearity for age, period, and cohort. A marked deviation from zero may indicate the presence of a cohort effect (nonlinearity of period and age effects). The age-period-cohort parameter, called the local drifts, is the APC (with two-tailed 95% CIs) of the expected age-specific rates over time. APC analysis was used to estimate the net drift parameter, which represents the net sum of the log-linear temporal trend arising from period effects and birth cohort effects. The 1-*df* Wald test was used to determine significance. *P* values less than 0.05 were considered statistically significant.

## Results

In Serbia during the period 1991–2010, nearly 42,000 people died of colorectal cancer; the average annual ASR was 14.2 per 100,000 people (Table 1). The average ASR of colorectal cancer mortality was higher in men (18.3 per 100,000) than in women (11.0 per 100,000).

**Table 1:** Colorectal cancer mortality in Serbia during the period 1991–2010: number of deaths, crude rate, and age-standardized rate (ASR)

Year	No. of deaths	Crude rate	ASR^a^
1991	1478	19.5	11.9
1992	1619	21.3	12.7
1993	1621	21.3	12.6
1994	1646	21.6	12.4
1995	1724	22.6	12.7
1996	1858	24.4	13.6
1997	1903	25.0	13.8
1998	1969	26.0	13.9
1999	1950	25.9	13.6
2000	2072	27.6	14.2
2001	2004	26.7	13.6
2002	2180	29.1	14.6
2003	2147	28.7	14.2
2004	2282	30.6	14.9
2005	2485	33.4	16.1
2006	2422	32.7	15.5
2007	2402	32.5	15.0
2008	2560	34.8	16.1
2009	2649	36.2	16.4
2010	2573	35.2	15.9
Total	41,544	27.8	14.2

^a^ASR was calculated by using Segi’s world standard population. All rates in this table are presented as the number of deaths per 100,000 people

**Fig. 1:**
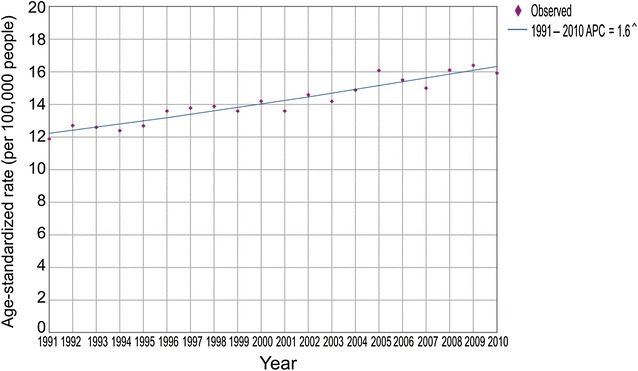
Trend in mortality of colorectal cancer in Serbia during the period 1991–2010: a joinpoint regression analysis. ^^^Statistically significant trend, *APC* average percentage change

**Fig. 2:**
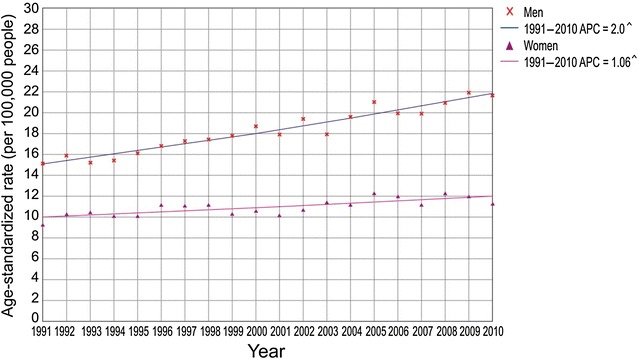
Trend in mortality of colorectal cancer in Serbia, by sex, during the period 1991–2010: a joinpoint regression analysis. ^^^Statistically significant trend, *APC* average percentage change

During the study period, we observed a significant trend of increase in colorectal cancer mortality (AAPC = 1.6%, 95% CI 1.3%–1.8%; Fig. 1). The colorectal cancer mortality trend increased in both men (AAPC = 2.0%, 95% CI 1.7%–2.2%) and women (AAPC = 1.0%, 95% CI 0.6%–1.4%; Fig. 2). The comparability test showed that the colorectal cancer mortality trends in men and women were not parallel (the final selected model rejected parallelism, *P* < 0.05).

In our study, the ASRs of people aged 50 years and older were 10–25 times higher than those of people in younger age groups (Table 2). Trends in age-specific mortality for colorectal cancer showed a significant increase for people aged 50 years and older (by 1.6, 2.1, 1.8, 1.5, 1.6, and 1.9% per year, respectively), whereas for people in younger age groups, non-significant changes were observed. Joinpoint analysis did not identify any joinpoint for the trend in any age group.

**Table 2:** Joinpoint regression analysis of colorectal cancer mortality in Serbia (by age) during the period 1991–2010, with data for the beginning and the end of the study period

Age (years)^a^	Average annual age-specific rate^b^	Year 1991		Year 2010		AAPC	95% CI
		No. of deaths	Mortality ^b^	No. of deaths	Mortality ^b^		
30–34	1.50	2	0.37	12	1.73	0.1	−4.3 to 4.8
35–39	2.84	31	4.85	16	3.25	−1.0	−2.8 to 0.9
40–44	6.28	36	6.19	33	6.96	0.3	−1.2 to 1.8
45–49	11.72	46	11.04	55	11.13	0.0	−1.2 to 1.3
50–54	20.70	87	16.93	128	24.13	1.6^c^	0.9 to 2.4
55–59	34.26	147	25.90	230	39.58	2.1^c^	1.6 to 2.7
60–64	56.88	223	42.40	286	61.34	1.8^c^	1.4 to 2.3
65–69	83.46	293	72.45	329	98.91	1.5^c^	1.1 to 1.9
70–74	120.96	190	96.10	451	128.23	1.6^c^	1.2 to 2.1
75–79	154.77	192	128.53	539	185.59	1.9^c^	1.4 to 2.4

*AAPC* average annual percentage change, *CI* confidence interval

^a^Joinpoint results are not shown for the subgroups of deceased people younger than 30 years because fewer than five colorectal cancer deaths occurred in each 5-year age group in any year

^b^All the rates are presented as number of deaths per 100,000 people

^c^Statistically significant trend

**Fig. 3:**
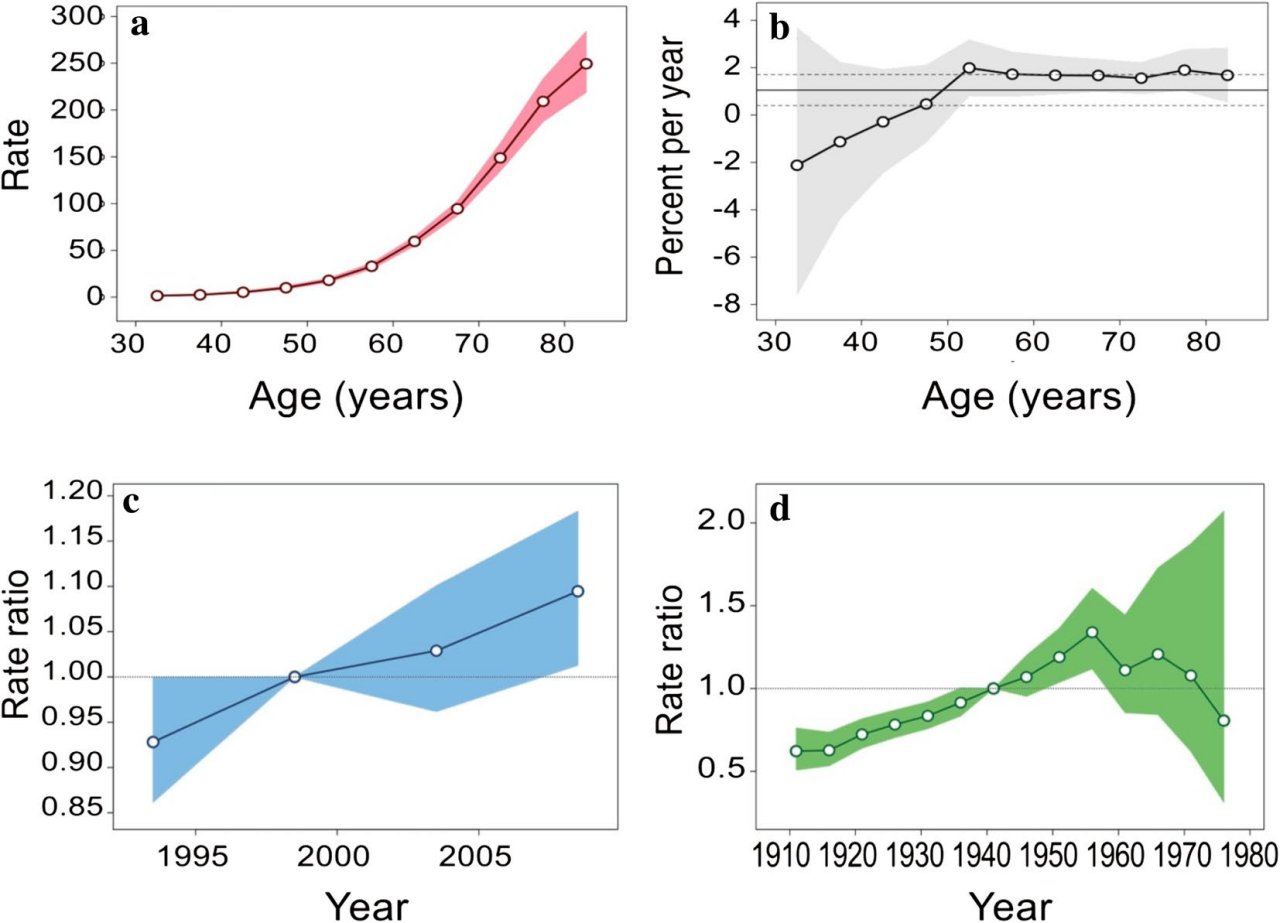
Mortality of colorectal cancer in Serbia during the period 1991–2010: an age-period-cohort analysis. **a** Longitudinal age curves of colorectal cancer mortalities: longitudinal age curves of the mortalities (per 100,000 people) of colorectal cancer and the corresponding 95% confidence intervals (the area colored in *pink*). **b** Local drift values for colorectal cancer mortalities: age group-specific annual percent change (%) in the mortalities of colorectal cancer and the corresponding 95% confidence intervals (the area colored in *grey*). **c** Period effects on colorectal cancer mortalities: period effects obtained from age-period-cohort analyses for the mortalities of colorectal cancer and the corresponding 95% confidence intervals (the area colored in *blue*). **d** Cohort effects on colorectal cancer mortalities: cohort effects obtained from age-period-cohort analyses for the mortalities of colorectal cancer and the corresponding 95% confidence intervals (the area colored in *green*)

Age-period-cohort analysis showed deviations from linearity for all three factors: age, period, and cohort (Table 3). These measures tended to distribute around approximately zero. A slight deviation from zero was observed for people born in the 1956–1960 cohort. In the Serbian population, the risk of death from colorectal cancer increased monotonically for people aged 30–79 years (Fig. 3). The local drift values were above zero in all of the oldest age groups (50–79 years), although a few insignificant exceptions were observed in the youngest age groups (30–39 years). The period effects remained relatively stable but were elevated in the most recent period (2001–2010). Colorectal cancer mortality increased for the first several birth cohorts, followed by downward flexion for people born after the 1960s. The local drifts were not statistically significant (Wald test = 7.943, *df* = 10, *P* = 0.634). Wald tests showed statistically significant cohort effects (*P* < 0.001) and non-significant period effects (*P* = 0.072).

**Table 3:** Age, period, and cohort effects on colorectal cancer mortality in Serbia during 1991–2010

Group	Effect	Deviation	95% Confidence interval
Age	30–34	−0.115	−0.410 to 0.180
	35–39	−0.181	−0.412 to 0.051
	40–44	0.011	−0.177 to 0.198
	45–49	0.099	−0.057 to 0.255
	50–54	0.115	−0.013 to 0.243
	55–59	0.155	0.056 to 0.253
	60–64	0.176	0.106 to 0.246
	65–69	0.068	0.012 to 0.124
	70–74	−0.048	−0.116 to 0.021
	75–79	−0.280	−0.380 to −0.180
Period	1991–1995	−0.009	−0.036 to 0.018
	1996–2000	0.017	−0.024 to 0.059
	2001–2005	−0.009	−0.047 to 0.029
	2006–2010	0.000	−0.026 to 0.026
Cohort	1916–1920	−0.169	−0.367 to 0.029
	1921–1925	−0.095	−0.216 to 0.027
	1926–1930	−0.063	−0.146 to 0.020
	1931–1935	−0.043	−0.101 to 0.014
	1936–1940	0.001	−0.058 to 0.060
	1941–1945	0.043	−0.038 to 0.125
	1946–1950	0.063	−0.045 to 0.172
	1951–1955	0.123	−0.006 to 0.252
	1956–1960	0.196	0.043 to 0.348
	1961–1965	−0.039	−0.228 to 0.150
	1966–1970	−0.002	−0.261 to 0.256
	1971–1975	−0.162	−0.572 to 0.248
	1976–1980	−0.499	−1.290 to 0.292

During the period 1991–2010 in Serbia, in men, overall mortality of colon cancer has been significantly increasing (by 2.6% per year), similar to that of rectal and anal cancer (by 1.4% per year), as shown in Tables 4 and 5. During the same period in Serbia, colon cancer mortality for women overall increased by 1.7% per year, but no significant changes for rectal and anal cancer mortality were observed (AAPC = 0.2%). In people of either sex who were 50 years of age or older, a significant increase in colon cancer mortality was observed. For men aged 50 years and above, rectal and anal cancer mortality significantly increased. For women in all age groups, no significant changes for rectal and anal cancer mortality were observed. According to the comparability test, overall mortality trends for colon cancer and rectal and anal cancer were parallel (the final selected model failed reject parallelism, *P* = 0.279).

**Table 4:** Number of deaths, crude and ASR of colorectal cancer mortality for men and women in Serbia during the period 1991–2010

Year	Men						Women					
	Colon cancer			Rectal and anal cancer			Colon cancer			Rectal and anal cancer		
	No. of deaths	Crude rate	ASR^a^	No. of deaths	Crude rate	ASR^a^	No. of deaths	Crude rate	ASR^a^	No. of deaths	Crude rate	ASR^a^
1991	370	10.0	6.9	452	12.2	8.2	314	8.1	4.4	342	8.8	4.9
1992	414	11.1	7.4	483	13.0	8.5	348	9.0	4.9	374	9.6	5.4
1993	390	10.5	6.9	467	12.6	8.3	403	10.4	5.6	361	9.3	4.9
1994	408	11.0	7.1	489	13.1	8.3	377	9.7	5.0	372	9.5	5.1
1995	430	11.5	7.2	525	14.1	8.9	383	9.8	5.0	386	9.9	5.1
1996	471	12.7	7.8	551	14.8	9.0	396	10.2	5.3	440	11.3	5.9
1997	495	13.3	8.1	560	15.1	9.2	451	11.6	5.9	397	10.2	5.2
1998	530	14.4	8.6	550	14.9	8.8	448	11.5	5.7	441	11.4	5.5
1999	526	14.3	8.2	603	16.4	9.6	432	11.2	5.5	389	10.0	4.8
2000	564	15.4	8.9	638	17.5	9.8	424	11.0	5.0	446	11.6	5.5
2001	556	15.2	8.7	606	16.6	9.2	411	10.7	5.0	431	11.2	5.2
2002	626	17.2	9.8	639	17.5	9.6	477	12.4	5.5	438	11.4	5.2
2003	564	15.5	8.5	617	17.0	9.4	470	12.2	5.5	496	12.9	5.9
2004	660	18.2	9.8	651	17.9	9.8	543	14.2	6.2	428	11.2	5.0
2005	688	19.0	10.2	719	19.9	10.8	601	15.7	6.8	477	12.5	5.6
2006	696	19.3	10.2	675	18.7	9.8	562	14.8	6.5	489	12.8	5.5
2007	719	20.0	10.1	673	18.8	9.8	535	14.1	6.1	475	12.5	5.1
2008	784	21.9	11.1	680	19.0	9.8	608	16.1	6.9	488	12.9	5.4
2009	781	21.9	10.8	774	21.7	11.1	615	16.4	6.6	479	12.7	5.4
2010	753	21.2	10.6	778	21.9	11.0	565	15.1	6.1	477	12.7	5.2
Overall	11,425	15.7	8.8	12,130	16.6	9.4	9363	12.2	5.7	8626	11.2	5.3

^a^ASR was calculated by using Segi’s world standard population. All rates in this table are presented as the number of deaths per 100,000 people

**Table 5:** Joinpoint regression analysis of age-specific mortality rates of colorectal cancer in Serbia, by sex, in the period 1991–2010

Age^a^	Colon cancer		Rectal and anal cancer	
	AAPC	95% CI	AAPC	95% CI
Men				
30–34	–^b^	–^b^	–^b^	–^b^
35–39	–^b^	–^b^	−3.3	−8.5 to 2.1
40–44	2.7^c^	0.3 to 5.1	−1.2	−4.5 to 2.3
45–49	−1.2	−3.5 to 1.2	0.6	−1.0 to 2.3
50–54	2.8^c^	1.4 to 4.3	1.5	−0.2 to 3.2
55–59	3.1^c^	2.1 to 4.1	2.1^c^	1.2 to 3.1
60–64	3.1^c^	2.1 to 4.2	2.0^c^	1.3 to 2.7
65–69	2.5^c^	1.7 to 3.3	1.5^c^	0.7 to 2.2
70–74	2.9^c^	2.0 to 3.8	0.7	−0.1 to 1.4
75–79	2.9^c^	1.9 to 3.8	1.8^c^	0.6 to 3.0
All men	2.6^c^	2.2 to 3.0	1.4^c^	1.1 to 1.7
Women				
30–34	–^b^	–^b^	–^b^	–^b^
35–39	1.7	−2.4 to 6.0	–^b^	–^b^
40–44	−0.8	−4.2 to 2.7	−0.1	−3.0 to 2.9
45–49	−0.2	−2.3 to 1.8	1.2	−1.6 to 4.0
50–54	2.3^c^	0.4 to 4.3	0.1	−1.9 to 2.2
55–59	2.4^c^	0.5 to 4.4	0.3	−0.8 to 1.4
60–64	2.2^c^	1.3 to 3.1	−0.7	−1.9 to 0.5
65–69	1.4^c^	0.5 to 2.4	−0.1	−0.8 to 0.7
70–74	1.6^c^	0.8 to 2.5	0.6	−0.2 to 1.5
75–79	1.7^c^	0.8 to 2.7	1.0	−0.1 to 2.2
All women	1.7^c^	1.1 to 2.3	0.2	−0.2 to 0.7

*AAPC* average annual percentage change, *CI* confidence interval

^a^ Joinpoint results are not shown for the subgroups of deceased people younger than 30 years, because fewer than five colorectal cancer deaths occurred in each 5-year age group in any year

^b^Joinpoint analysis was not possible, because there were no colorectal cancer deaths in at least one year during the observed period

^c^Statistically significant trend

## Discussion

The colorectal cancer mortality in Serbia increased considerably over the past two decades. Mortality increased particularly in men, but the trends were different according to age group and subsite. The increased colon cancer mortality among younger men is particularly worrisome. The exceptions were women, in whom no significant trend in rectal and anal cancer mortality was observed.

Worldwide in 2010, Hungary had the highest colorectal cancer mortalities for both men and women (31.1 per 100,000 men and 16.1 per 100,000 women); Georgia and Egypt had the lowest mortalities for both sexes (approximately 2.1 per 100,000 men and 1.6 per 100,000 women) [3]. Serbia was among the countries with high colorectal cancer mortality; other countries with high mortality included the Russian Federation, Poland, Portugal, Slovenia, and the Czech Republic. Except in only a few countries (Kuwait, Cuba, and Qatar), the colorectal cancer mortality was higher for men than for women [3]. The large geographic differences in the global distribution of colorectal cancer are generally difficult to explain [6]. The high mortality across central and eastern European countries, as well as in Serbia and some Mediterranean countries, likely reflects fundamental changes that occurred during the transition period since the 1980s [22]. These high mortalities are most likely the result of increased risk factors associated with “westernization,” such as increased animal fat and red meat consumption, low vegetable intake, obesity, physical inactivity, tobacco use, and alcohol consumption, which, in the past few decades, characterized newly economically developed countries [5, 10]. In our study, we found that colorectal cancer mortality increased markedly with age in the cohort of people born between 1956 and 1960. This finding must be interpreted carefully, though, because these cohort values are based on fewer deaths (i.e., lower ASRs). However, since trends in birth cohort effects usually reflect risk factor trends, this increase seems to indicate an increasing colorectal cancer risk in this cohort. During this 1991–2010 period (after the separation from the Eastern Bloc), Serbia sought to accelerate economic development, urbanization, and industrialization, which led to changes in lifestyle and dietary structure. Social circumstances in Serbia were further affected by a devastating economic crisis, which was exacerbated by United Nations sanctions, civil wars in the former Yugoslavia (1991–1995), hundreds of thousands of refugees, the collapse of the dinar, the inability to purchase needed medications, the deterioration of public health, and the bombing of Serbia (1999), as well as post-2000 changes during democratization [23, 24]. In addition, the mortality partly reflects varying data quality worldwide [25].

During the last decade, the colorectal cancer mortalities for both men and women annually declined in the United States (by 2.9% per year for all races) [4] and in most countries of Western and Northern Europe (in Germany by 2.0%, France by 1.7%, and the United Kingdom and Italy by 1.5% per year) [5]. Conversely, the colorectal cancer mortalities for both men and women have shown a continuous annual rise over the last decade in some Eastern and Central European countries, such as Russia (by 0.6% per year) and Croatia and Serbia (by 1.6% per year) [5, 26]. Also, increased trends have been observed in some Central and South American countries (e.g., Mexico and Brazil) [27]. On the other hand, in Slovakia and Slovenia the colorectal cancer mortalities were very high, but they remained constant for both men and women in the last decade [6]. In most countries, colorectal cancer mortality trends for both men and women were more favorable in young people (aged under 50 years) [6]. In Spain, Poland, and Hungary, during the period 1970–2007, particularly favorable colorectal cancer mortality trends were observed in women in all age groups compared to men [6]. The decrease in colorectal cancer mortality in the United States and Western Europe (e.g., the United Kingdom and France) could be attributed to long-term screening programs and improvements in treatment protocols, as well as to positive lifestyle changes [7–9]. In some Eastern European countries, the benefits, however, of short-term colorectal cancer screening, improvements in treatment, and recent positive changes in dietary and lifestyle habits are still only estimated [9–11, 28]. The promising colorectal cancer mortality trends in women and young people may reflect recent positive changes in diet and lifestyle habits (such as reduced alcohol drinking and tobacco smoking), which have been recommended as cancer prevention measures [11, 29].

In Serbia, the lack of decline in colorectal cancer mortality indicates suboptimal levels of cancer control. In 2013, a national program for the early detection of colorectal cancer was implemented. Countrywide data are not yet available, but a single-institution analysis found that the 5-year overall survival rate was 57.8% in patients younger than 40 and 28.5% in patients over 65 years of age [30]. For patients with colorectal cancer in Europe, EUROCARE-4 study investigators reported a 5-year relative survival rate of 53.8%; in this study, the highest survival rates were observed in the Nordic and Central European countries, and the lowest survival rates were observed in Eastern Europe [31]. In central Serbia during the period 1999–2008, colorectal cancer incidence was high and showed an increasing trend [22, 25]. The analysis of disease burden in Serbia showed that, for colorectal cancer, the harmful effects of physical inactivity were higher in women than in men (31.0% vs. 20.6% of total disability-adjusted life years [DALY]), as were the harmful effects of being overweight (16.3% vs. 13.1% of total DALY) [32]. The 2006 National Health Survey found that, in Serbia, 54.5 % of people were overweight, subdivided as 18.3% obese and 36.2% pre-obese [33]. This survey also found that the average body mass index (BMI) of Serbians older than 20 years was 26.7 kg/m^2^ (27.4 for men and 26.0 for women); this represented a substantial increase over 2000, when the average BMI was 26.0. In 2006, nearly one-third of the employed population of Serbia (31.1%) had a sedentary type of work; this, too, represented a substantial increase over 2000, when the figure was only 25.2%. Also, more than two-thirds of Serbian people (67.7%) spent their free time mainly in a sedentary manner. In 2006 in Serbia, smoking was more prevalent in men (38.1%) than in women (29.9%). On average, men drank four times more alcohol than women (8.5 vs. 2.0 drinks per week) [33]. Similarly, in 30 European countries, the level of exposure to underlying lifestyle cancer risk factors corresponds to the pattern of cancer incidence [34]. In the present study, different time trends in mortality for both sexes and for subsites in the Serbian population suggest that colorectal cancer has, at least in part, different etiological factors. Furthermore, the possibility that proximal colon cancers differ from distal colon cancers in terms of biology or carcinogenesis could be an explanation for some variations between countries in the epidemiological and clinical peculiarities of colorectal cancer, as well as differences in cancer detection, treatment, and survival [35, 36].

A strength of our study is that it provides the first nationwide estimates of colorectal cancer mortality in Serbia in last two decades. Another strength is that it is a population-based study that used high-quality cancer data with temporal trends analyzed by both joinpoint and age-period-cohort analysis. Also, this high-quality data (including a comprehensive, countrywide death registration system in Serbia) enabled us to make comparisons with other countries [25, 37]. Regarding causes of death in Serbia, the World Health Organization (WHO) assessed the quality of data as moderate [37]. For example, for the most recent year (2012), the percentage of unknown and ill-defined cancer deaths in Serbia was 3.8%, which the WHO considered as moderate quality [25]. Contrary to the trends in many countries, the colorectal cancer mortality in Serbia is increasing. Because of this rising mortality trend in Serbia, more effective cancer prevention measures (such as measures focusing on diet and the control of healthy weight, tobacco use, and alcohol consumption), more effective treatments for colorectal cancer, and more effective cancer screening programs focusing on early diagnosis are needed.

Our study did have several limitations. First, we acknowledge that a longer study period may have enabled us to better assess mortality trends, but in Serbia no data were available for this. Second, no separate data exist on colorectal cancer deaths among refugees that can possibly confound the colorectal cancer pattern in Serbia. Clearly, a higher-quality death registration system, which is available in wealthier countries, is needed in Serbia. Third, during the study period, two different revisions of the ICD were used for cancer coding. Fourth, no data are available on colorectal cancer incidence for the entire Republic of Serbia, which limited our ability to explain the increasing mortality trends. Fifth, we had no reliable data on colorectal cancer therapy in Serbia during the study period. The available literature does not have enough relevant information concerning colorectal cancer risk factors in the Serbian population. Fecal occult blood testing has been recommended as a national colorectal cancer screening test since only 2013. Therefore, the incompleteness of colorectal cancer screening data in Serbia, which could be used to explain mortality trends, is a limitation of our study. Future studies should investigate whether patterns of risk factors are present in some age cohorts. The colorectal cancer mortality in Serbia contributes to its ranking among countries with the highest mortality. Increasing trends in the colorectal cancer mortality indicate that improved primary and secondary prevention measures, which target young men, particularly, are needed. Reduction in the burden of colorectal cancer in Serbia will require the implementation of a comprehensive national screening program.
